# CPEB1 mediates hepatocellular carcinoma cancer stemness and chemoresistance

**DOI:** 10.1038/s41419-018-0974-2

**Published:** 2018-09-20

**Authors:** Min Xu, Shiji Fang, Jingjing Song, Minjiang Chen, Qianqian Zhang, Qiaoyou Weng, Xiaoxi Fan, Weiqian Chen, Xulu Wu, Fazong Wu, Jianfei Tu, Zhongwei Zhao, Jiansong Ji

**Affiliations:** 1 Key Laboratory of Imaging Diagnosis and Minimally Invasive Intervention Research, The Fifth Affiliated Hospital of Wenzhou Medical University, 323000 Lishui, China; 2 Department of Radiology, Affiliated Lishui Hospital of Zhejiang University, 323000 Lishui, China

## Abstract

Cancer stem cells (CSCs) are a subpopulation of cells within tumors that are believed to possess pluripotent properties and thought to be responsible for tumor initiation, progression, relapse and metastasis. Cytoplasmic polyadenylation element-binding protein 1 (CPEB1), a sequence-specific RNA-binding protein that regulates mRNA polyadenylation and translation, has been linked to cancer progression and metastasis. However, the involvement of CPEB1 in hepatocellular carcinoma (HCC) remains unclear. In this study, we have demonstrated that CPEB1 directly regulates sirtuin 1 (SIRT1) mRNA to mediate cancer stemness in HCC. Cancer stemness was analyzed by self-renewal ability, chemoresistance, metastasis, expression of stemness-related genes and CSC marker-positive cell populations. The results indicate that CPEB1 is downregulated in HCC. Overexpression of CPEB1 dramatically reduced HCC cell stemness, whereas silencing CPEB1 enhances it. Using site-directed mutagenesis, a luciferase reporter assay, and immunoprecipitation, we found that CPEB1 could directly target the 3′-UTR of SIRT1, control poly(A) tail length and suppress its translation to mediate cancer stemness in vitro and in vivo. Overall, our findings suggest that the negative regulation between CPEB1 and SIRT1 contributes to the suppression of cancer stemness in HCC. CPEB1 may have potential as a therapeutic target in HCC.

## Introduction

The incidence of hepatocellular carcinoma (HCC) has been increasing worldwide owing in part to extrinsic factors such as chronic liver disease caused by viral infections, alcohol and nonalcoholic fatty liver disease^1–4^. HCC is also associated with a high mortality because of its prolific rate of recurrence and heterogeneity, which has been attributed to the existence of cancer stem cells (CSCs)^5^. The proliferation and differentiation capabilities of liver CSCs are believed to be responsible for tumor initiation, progression, relapse, metastasis and resistance to therapy^6,7^. For this reason, CSCs and their associated pathways are becoming the focus of potential therapies for HCC.

The heterogeneity of HCC has previously been attributed to hepatocytes because the liver is thought to lack a defined stem cell population for organ maintenance^8^. However, growing evidence indicates that a distinct subpopulation of cells in liver tumors exhibit properties that are consistent with stemness^9,10^. Furthermore, high expression levels of CSC markers, such as OCT4, NANOG, SOX2 and LIN28, have been found in subpopulations of some HCC cell lines^11,12^. Cells in these subpopulations have a spheroid morphology and are strongly associated with invasive ability, self-renewal and chemoresistance^13^. Recently, the RNA-binding protein Musashi 2 (MSI2), which is a potent oncogene in myeloid leukemia and gastrointestinal malignancies, was found to enhance CSC properties, including self-renewal, drug resistance and tumorigenicity, by activating LIN28 in a mouse xenograft model of HCC^14^. MSI2 is one of several RNA-binding proteins that are known to be involved in cytoplasmic polyadenylation^15,16^.

Cytoplasmic polyadenylation element-binding protein 1 (CPEB1) is another protein involved in cytoplasmic polyadenylation that may influence tumorigenesis. CPEB1 anchors the non-canonical poly(A) polymerases Gld2 or Gld4, as well as the deadenylating enzyme PARN (poly(A) ribonuclease), to bind to cytoplasmic polyadenylation elements (CPEs) found in the 3′-untranslated region (UTR) of specific mRNAs^17,18^. This regulates poly (A) tail growth or removal, which consequently promotes or represses translation. It is also particularly important for regulating mRNAs that participate in the G2–M transition of the cell cycle^19,20^. Reduced levels of CPEB1 are associated with several types of cancer, cell invasion and angiogenesis^21^. CPEB1 knockdown causes some metastasis-related mRNAs to have shorter or longer poly(A) tails. CPEB1 levels are known to decrease when breast cancer cells become metastatic^22^. Moreover, strong evidence indicates that CPEB1 modulates the differentiation of glioma stem cells and restrains the proliferation of glioblastoma cells^23,24^. However, the involvement of CPEB1 in HCC remains unclear, and its roles in HCC cancer stemness, self-renewal and chemoresistance is yet to be elucidated.

In this work, we explored the characteristics and roles of CPEB1 in HCC cell lines and HCC tumor tissue. We also assessed the possibility that CPEB1 directly regulates sirtuin 1 (SIRT1) to mediate cancer stemness in HCC through an interaction with a CPE site. Finally, we determined whether CPEB1 could attenuate tumor growth and chemoresistance in vivo using a mouse model.

## Materials and methods

### Cell lines and cultures

Human HCC cell lines HepG2, Huh7 and SK-Hep1, a normal human hepatic cell line (L02) and HEK293T cells were all purchased from the Shanghai Institute of Cell Biology, Chinese Academy of Sciences (Shanghai, China). The metastatic human HCC cell line MHCC-LM3 was from the Liver Cancer Institute, Zhongshan Hospital, Fudan University (Shanghai, China). Cells were maintained in Dulbecco’s modified Eagle’s medium (DMEM; Gibco, Carlsbad, CA, USA) with 10% heat-inactivated fetal bovine serum (FBS, Gibco), 1% penicillin (100 U/ml) and 0.1 mg/ml streptomycin (Solarbio, Beijing, China) in a humidified chamber with 5% CO_2_ and 95% air at 37 °C.

### RNA extraction and Real-time quantitative PCR (qRT-PCR)

Total RNA from tissues or cells was extracted using Trizol reagent (Invitrogen, Grand Island, NY, USA). Primer sequences used in this study are listed in Table 1. Complementary DNA (cDNA) was synthesized using a reverse transcriptase kit (Invitrogen) under the following PCR conditions: 95 °C for 30 s; 95 °C for 5 s and 60 °C for 34 s (40 cycles); then 72 °C extension for 5 min. The mean from three independent experiments was used to quantify the RNA.

**Table 1:** Sequences of qRT-PCR primers used in this study

Gene	GenBank no.	Primer sequence (5′-3′)	Product length
CPEB1	NM_001079533	Forward: GTCCTCCCAAAGGTAATATGCC	262
		Reverse: TGCAGAGCACCGACAAACA	
SIRT1	NM_001142498	Forward: CCCCATGAAGTGCCTCAGAT	223
		Reverse: TGGGTGGCAACTCTGACAAA	
CD133	NM_001145847	Forward: TCACCAGCAACGAGTCCTTC	270
		Reverse: GGTTTGCACGATGCCACTTT	
CD24	NM_001291737	Forward: GCTCCTACCCACGCAGATTT	162
		Reverse: GAGACCACGAAGAGACTGGC	
EpCAM	NM_002354	Forward: CCATGTGCTGGTGTGTGAAC	159
		Reverse: GAAGTGCAGTCCGCAAACTT	
OCT4	NM_001173531	Forward: ATGTGGTCCGAGTGTGGTTC	232
		Reverse: GAGACAGGGGGAAAGGCTTC	
NANOG	NM_001297698	Forward: AGACAAGGTCCCGGTCAAGA	246
		Reverse: AGGCATCCCTGCGTCACAC	
SOX2	NM_003106	Forward: TTTGTCGGAGACGGAGAAGC	237
		Reverse: TAACTGTCCATGCGCTGGTT	
LIN28	NM_024674	Forward: ACCGGACCTGGTGGAGTATT	199
		Reverse: GCGGACATGAGGCTACCATA	
GAPDH	NM_001256799	Forward: GAGAAGGCTGGGGCTCATTT	231
		Reverse: AGTGATGGCATGGACTGTGG	

### Western blot analysis

Proteins were extracted from washed cells and homogenized tumor tissues with lysis buffer (100 μl/50 ml). Equal amounts of protein sample were separated by sodium dodecyl sulfate–polyacrylamide gel electrophoresis and transferred to polyvinylidene fluoride (PVDF) membranes (Millipore, Bedford, MA, USA). Membranes were incubated overnight at 4 °C with primary antibodies anti-CPEB1 and anti-SIRT1 (Abcam, Cambridge, MA, USA); and antibodies for OCT4, NANOG, SOX2, LIN28 and GAPDH (Santa Cruz Technology, Santa Cruz, CA, USA). Membranes were then thoroughly washed with Tris-buffered saline–Tween 20 (TBST) and incubated for 1 h at room temperature with horseradish peroxidase-labeled secondary antibody (Santa Cruz Technology, Santa Cruz, CA, USA). After washing in TBST, the membranes were visualized with an enhanced chemiluminescence (ECL) system. The mean values from three experiments were obtained.

### Cell proliferation assay

Stably or transiently transfected cells were plated in 96-well plates at a concentration of 2000 cells/well in complete medium. At 24, 48 and 72 h time points, cell proliferation assessment was carried out using a Cell Counting Kit 8 (CK04-20; Dojindo, Kumamoto, Japan), according to the manufacturer’s protocol.

### Sphere formation assay

HCC cells (1 × 10^3^) were plated onto six-well poly HEMA-coated plates (Sigma-Aldrich, St. Louis, MO, USA) and cultured in sphere medium containing DMEM/F12 medium (Invitrogen, Carlsbad, CA, USA) supplemented with 4 μg/mL insulin (Sigma), B27 (Invitrogen), 20 ng/mL EGF (Sigma) and 20 ng/mL basic FGF (Invitrogen) for 10 days. Spheroids were counted under a microscope at ×200 magnification and representative fields were photographed.

### Patients and tissue samples

Tumor biopsies and corresponding adjacent tissues were collected from HCC patients who underwent surgery at Lishui Central Hospital, Zhejiang Sheng, China. This study was conducted in accordance with the Declaration of Helsinki and approved by the Ethics Committee of Lishui Central Hospital. All study participants gave written informed consent. Commercial tissue microarrays (TMAs) were obtained from Shanghai Bio-Chip Co. Ltd. (Shanghai, China). The HCC TMA used in the present study contained 68 primary HCC and 60 adjacent non-cancerous liver tissues, with the age of the donors ranging between 18 and 73 years (mean age, 48.31 years). To investigate the expression of CPEB1 at the mRNA level, a large cancer dataset with high-throughput sequencing data for protein-coding genes (mRNA), which included 371 primary HCC tissues, was downloaded from The Cancer Genome Atlas (TCGA). The detailed information regarding the clinical features of the patients is presented in Table 2.

**Table 2:** Clinicopathological characteristics of patients with HCC and their associations with CPEB1 expression

Clinical feature	Case number (*n*)		CPEB1 expression (*n*, %)		*P*-value	Liver TCGA dataset	SIRT1 expression (*n*, %)		*P*-value
	Biopsies	TMAs	Low	High			Low	High	
Total no. of cases	12	68	80			371	80		
Gender					0.251				0.462
Male	8	58	37 (56.1)	29 (43.9)		245	31 (47.0)	35 (53.0)	
Female	4	10	6 (42.9)	8 (57.1)		117	6 (42.9)	8 (57.1)	
Age					0.043				0.73
<60	3	56	34 (57.6)	25 (42.4)		167	27 (45.8)	32 (54.2)	
≥60	9	12	12 (57.1)	9 (42.9)		191	10 (47.6)	11(52.4)	
Clinical stage					0.037				0.052
I/I–II	2	4	2 (33.3)	4 (66.7)		168	3 (50)	3 (50)	
II/II–III	7	22	17 (58.6)	12 (41.4)		84	13 (44.8)	16 (55.2)	
III	3	41	25 (56.8)	19 (43.2)		82	21 (47.7)	23 (52.3)	
III–IV	0	1	1 (100)	0 (0)		6	1 (100)	0 (0)	
pT stage					0.082				0.101
T1–T2	7	34	20 (48.8)	21 (51.2)		227	22 (53.7)	19 (46.3)	
T3–T4	5	34	21 (53.8)	18 (46.2)		130	19 (48.7)	20 (51.3)	
Serum AFP (ng/ml)					0.754				0.253
<25	6		3 (50)	3 (50)			2 (33.3)	4 (66.7)	
≥25	6		2 (33.3)	4 (66.7)			4 (66.7)	2 (33.3)	
Tumor size (cm)^a^					0.483				0.116
<5	8		5 (62.5)	3 (37.5)			4 (50)	4 (50)	
≥5	4		2 (50)	2 (50)			2 (50)	2 (50)	
Liver cirrhosis					0.079				0.034
Yes		53	25 (47.2)	28 (52.8)			26 (49.1)	27 (50.9)	
No		15	9 (60)	6 (40)			7 (46.7)	8 (53.3)	
HBV					0.165			0.072	
Yes		31	17 (54.8)	14 (45.2)			17 (54.8)	14 (45.2)	
No		37	20 (54.1)	17 (45.9)			18 (48.6)	19 (51.4)	
Vascular invasion					0.265				0.025
Yes		16	7 (43.8)	9 (56.2)			7 (43.8)	9 (56.2)	
No		52	28 (53.8)	24 (46.2)			25 (48.1)	27 (51.9)	

^a^The largest dimension of the tumor specimen

### Cell migration assays

Cells (50,000 per well) were seeded on the top of 24-well Transwell plates (Sigma-Aldrich, St Louis, MO, USA) coated with or without Matrigel (BD Biosciences, San Jose, CA, USA). Cells were grown in DMEM containing 5 ng/ml transforming growth factor-β and allowed to migrate and invade for 24 h. Photographs of five randomly selected fields of the fixed and crystal violet-stained cells were captured and cells that passed to the lower surface were counted. Experiments were repeated independently three times.

### Flow cytometric analysis

HepG2 or MHCC-LM3 cells were first labeled with primary antibody for CD133 (Miltenyi Biotec, Bergisch Gladbach, Germany) and then incubated with goat anti-mouse IgG microbeads (Miltenyi Biotec) according to the manufacturer’s protocols. Cells were then sorted magnetically using MACS LS columns (Miltenyi Biotec). After dead cells were excluded from the sort via an electronic gate, cells expressing CD133 were collected through a sort gate. In addition, phycoerythrin (PE)-conjugated CD133 (BD PharMingen, San Jose, CA, USA) were used in the experiment. The processed cells were incubated in phosphate-buffered saline (PBS) containing 2% FBS followed by PE-conjugated antibodies. Isotype-matched mouse immunoglobulins served as controls. The samples were analyzed using a FACSCanto II analyzer flow cytometer (BD Biosciences, San Jose, CA, USA).

### TUNEL assay

Terminal deoxynucleotidyl transferase-mediated dUTP nick end labeling (TUNEL) staining was performed to assess in situ DNA fragmentation using a commercial kit (ApopTag Kit-S7100, Chemicon, Temecula, CA, USA) following the manufacturer’s protocol. The incidence of apoptosis in each subgroup was quantified by counting the number of TUNEL-positive cell nuclei under a Nikon ECLIPSE Ti fluorescence microscope (×400 magnification) and photographed with a CoolSNAP photometric camera. The number of apoptotic cells was determined as the mean of 10 areas from each preparation.

### Colony formation assay

To assess colony formation, doxorubicin (5 μg/ml) or dimethylsulfoxide (DMSO) was added to cells seeded onto six-well plates (1000 cells per well). After 14 days, the colonies were fixed with methanol and then stained with 0.5% crystal violet in 20% methanol for 15 min and counted. Representative wells were photographed.

### RNA immunoprecipitation assay

After cells were washed with cold PBS, they were lysed with RNA immunoprecipitation (RIP) lysis buffer (EMD Millipore, Billerica, MA, USA) according to manufacturer’s instructions. The lysate was then incubated with antibody/beads for 18 h at 4 °C. The resultant immunoprecipitated CPEB1–RNA complexes were washed and treated with proteinase K and recovered by phenol–chloroform extraction followed by ethanol precipitation. RT-PCR analysis was then conducted.

### PCR poly(A) tail (PAT) assay

Total cellular RNA was reversed transcribed with MultiScribe reverse transcriptase (Life Technologies), using oligo(dT) anchor primer (5′-CCAGTGAGCAGAGTGACGAGGACTCGAGCTCAAGCTTTTTTTTTTTTTTTTT-3′), and subsequent PCR was conducted with anchor primer (5′-CAGAGTGACGAGGACTCGAG-3′) and specific primer for SIRT1 (5′-GTAGACTGTTTAATGACTGG-3′) located near the 3′ end of the SIRT1 3′-UTR.

### Lentivirus production and transduction

Lentivirus vectors containing the DNA fragment and short hairpin RNA (shRNA) against CPEB1 (shCPEB1) and the negative control (Scramble) were constructed and generated by Gene Pharma (Shanghai, China). The sequences of small interfering RNA (siRNA) or shRNA used for siRNA transfections were shCPEB1-#1: 5′-UGAGGAAUCUGAGUCCUGGGU-3′ and shCPEB1-#2: 5′-AUCUGAUCCAGAGCUGAAGCC-3′. For infection, the media containing retrovirus was added to the cells supplied with polybrene (8 μg/mL) for 6 h and then replaced with fresh medium. Then, after 12 h, the infection was repeated to obtain stable cell lines. Transfections were performed by using Lipofectamine 2000 reagent (Invitrogen) and plasmid DNA or siRNAs in Opti-MEM I (Invitrogen) following manufacturer’s instructions. To monitor transfection efficiency, DNA was co-transfected at a ratio of 1:10 with the reporter plasmid pEGFP-C3 (Clontech) and siRNAs were co-transfected with a fluorescent siRNA control at a ratio of 1:10. Cells were analyzed by fluorescence microscopy after 48-h incubation.

### Luciferase reporter assay

HCC cells were seeded in a 12-well plate and transfected with plasmids. The SIRT1 3′-UTR luciferase reporter vector was constructed by Genechem (Shanghai, China). Site-directed mutagenesis was performed using the QuickChange Lightning kit (Stratagene, La Jolla, CA, USA). Sequencing was used to confirm the correct mutations had been generated. After co-transfecting with CPEB1 or shCPEB1 and the corresponding mock for 48 h, MHCC-LM3 and HepG2 cells were harvested and assayed with a dual-luciferase assay (Promega) according to the manufacturer’s instructions. Cells were transfected with reporter vectors and the Renilla luciferase was used as a control and for normalization.

### Animal experiments

Male nude mice (6–8 weeks old) were purchased from the Chinese Science Academy (Shanghai, China). All animal studies were approved by the Animal Care and Use Committee. MHCC-LM3 cells (1 × 10^6^) transfected with either CPEB1 or a control vector were injected subcutaneously into the right axilla of each nude mouse to create an HCC model. Doxorubicin (1 mg/kg) was orally administered once every 2 days for six doses starting 14 days after tumor implantation. The xenograft tumor size was monitored every 3 days (volume = width^2^ × length × 1/2). Mice were euthanized at the end of the experiment and the tumors were excised. Tumors were fixed in 10% formalin, embedded in paraffin, and cut into 4 μm thick slices.

### TMA and immunohistochemistry

TMA sections were stained with an automatic immunohistochemical staining device (Benchmark XT; Ventana Medical Systems, Tucson, AZ, USA) and visualized with an OptiView DAB IHC Detection Kit (Ventana Medical Systems) according to the manufacturer’s instructions. The slides of paraffin-embedded xenograft tissues were probed with the same primary antibodies used for western blotting and anti-ki67 (Abcam). The staining processes were performed as previously described^25^ and quantified with Image ProPlus (IPP) software (Media Cybernetics, Rockville, MD, USA). Slides were counterstained with hematoxylin and analyzed under a microscope (BX51; Olympus, Tokyo, Japan). The staining levels were scored as 0 (negative), 1 (weakly positive), 2 (moderately positive) or 3 (strongly positive).

### Statistical analysis

Data were analyzed using SPSS software (version 16). Each experiment was performed in triplicate and values are presented as mean ± SD. The two-tailed Student’s *t*-test was used to analyze statistical differences between groups. *P*-values < 0.05 were considered statistically significant.

## Results

### Expression of CPEB1 is low in liver cancer cells and downregulated in liver cancer tissue

**Fig. 1:**
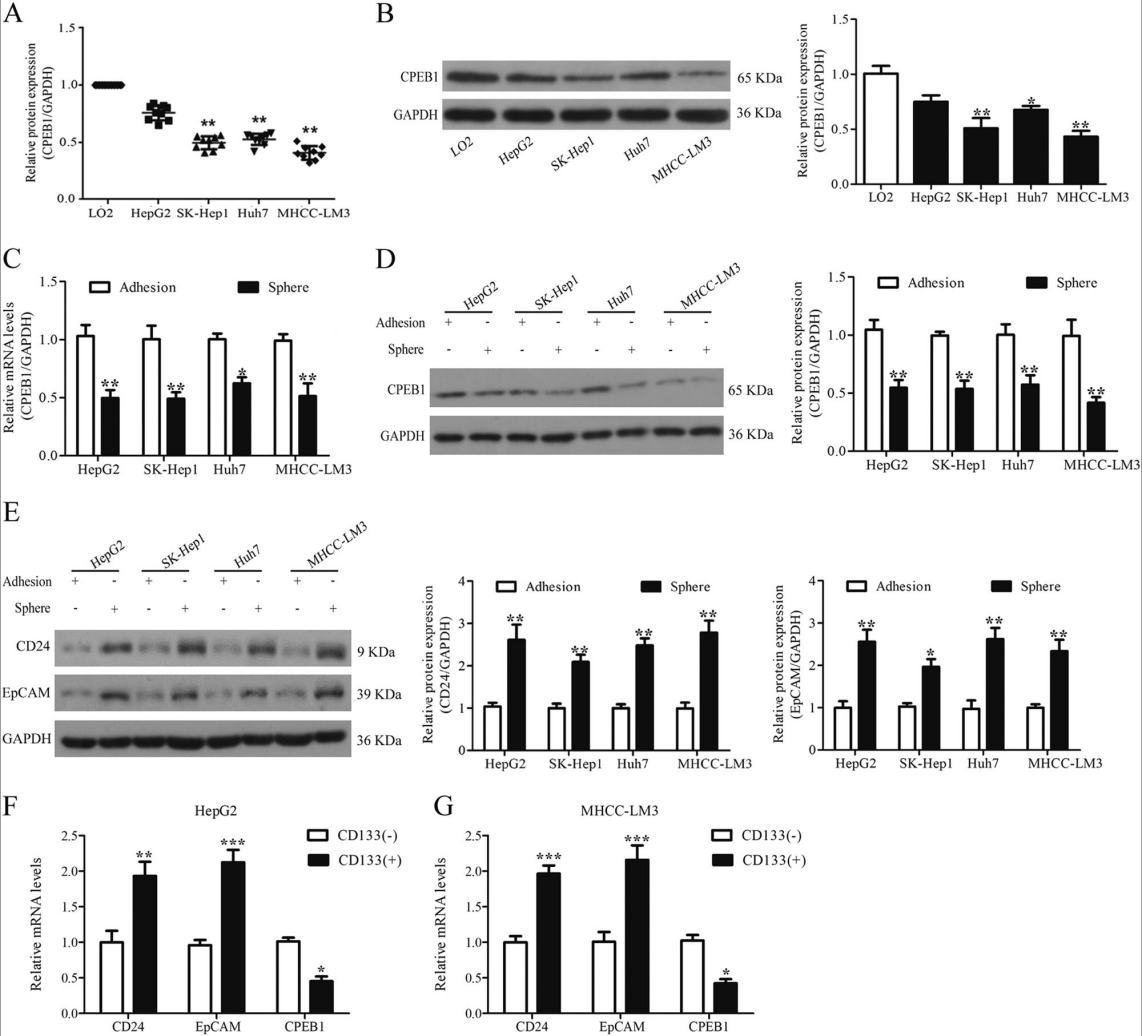
CPEB1 expression is lower in liver cancer cell lines. **a**, **b** CPEB1 expression is decreased in four HCC cell lines (HepG2, Huh7, SK-Hep1 and MHCC-LM3) compared with normal hepatic cells (LO2) by real-time PCR and western blot analysis. MHCC-LM3 showed the lowest levels of CPEB1 expression. GAPDH was used as a loading control. **c**, **d** CPEB1 expression levels were also tested in spheroid cells and the corresponding adherent cells by quantitative RT-PCR and immunoblotting. The levels in spheroid cells, which are considered cells to have stem cell characteristics, were lower than in adherent cells, whereas CD24 and EpCAM levels were higher (**e**). **f**, **g** CPEB1 expression levels were verified in HepG2 and MHCC-LM3 cells with a CD133 marker sorted by MACS and a the two markers CD24 and EpCAM by quantitative RT-PCR. CPEB1 expression was lower in CD133^+^ cells in comparison with their counterpart CD133^−^cells at the mRNA level. The data are presented as the means ± SEM. *P < 0.05, **P < 0.01, ***P < 0.001

A reduction of CPEB1 expression is associated with the progression of various cancers. Therefore, we first compared the expression of CPEB1 in HCC cell lines (HepG2, Huh7, SK-Hep1 and MHCC-LM3) with that in normal hepatic cells (LO2). The results, obtained by quantitative PCR (qPCR) and immunoblotting, indicate that CPEB1 expression and protein levels are lower in HepG2, Huh7, SK-Hep1 and MHCC-LM3 than in control cells with the lowest expression found in MHCC-LM3 cells (Fig. 1a, b). CPEB1 expression and protein levels were assessed in spheroid and adherent cells from each HCC cell line by qPCR and immunoblotting (Fig. 1c, d). The expression of CPEB1 is predicted to be lower in spheroids, which were thought to have stem cell characteristics, compared with the corresponding adherent cells. Additionally, CD24 and EpCAM expression were also verified in corresponding spheroid and adherent cells (Fig. 1e). HepG2 and MHCC-LM3 cells were then sorted by the presence of the hematopoietic stem cell marker CD133. CPEB1 expression was verified as being significantly reduced in cells that were positive for CD133 expression, whereas CD24 and EpCAM expression were significantly increased (Fig. 1f, g). These results confirm that CPEB1 expression is lower in the CSCs of HCC cell lines.

**Fig. 2:**
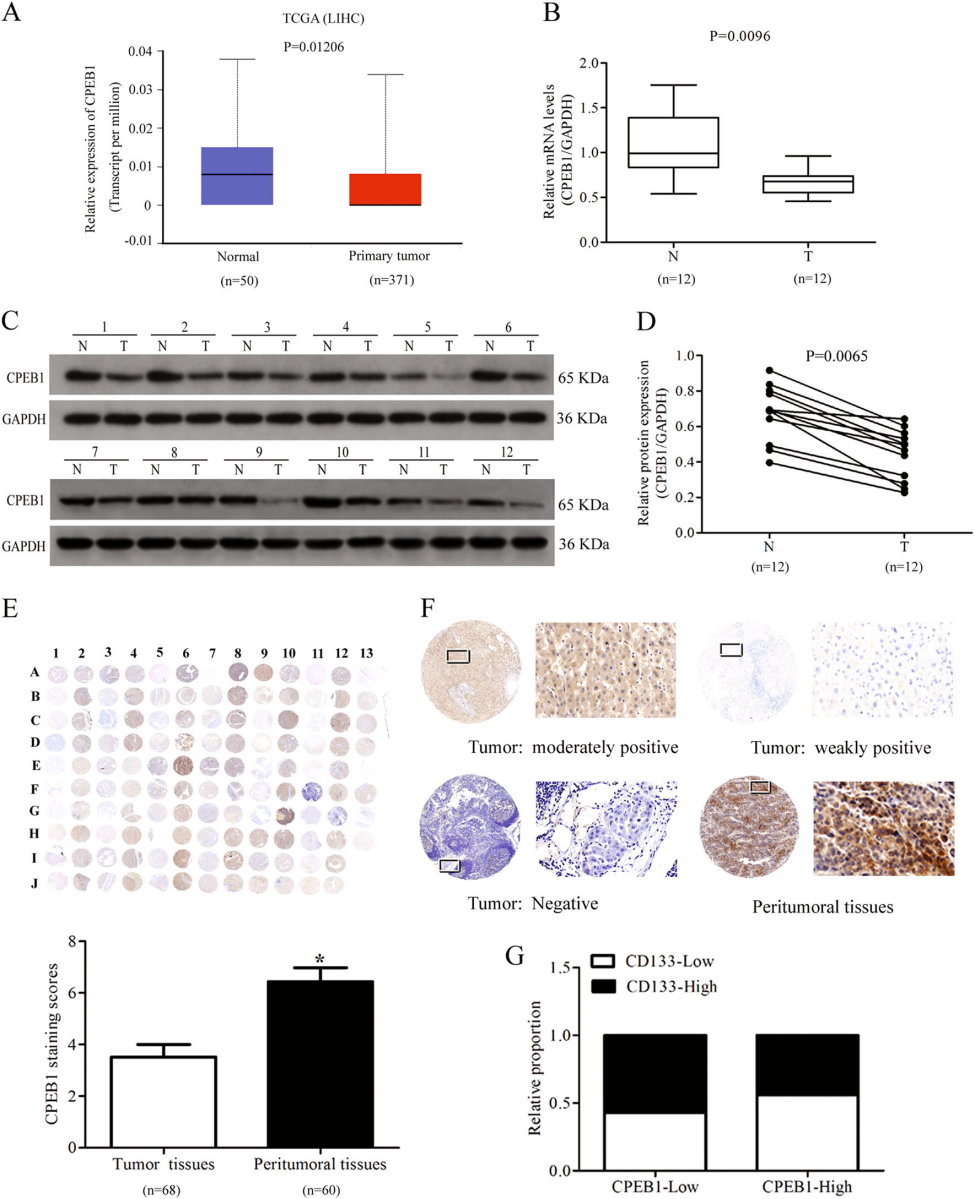
CPEB1 is downregulated in liver cancer tissue. **a–d** CPEB1 levels, detected by quantitative RT-PCR and immunoblotting, were significantly downregulated in data from The Cancer Genome Atlas (TCGA) and human HCC patient samples (T: tumor tissues, N: adjacent non-tumor tissues). **e** Immunohistochemical staining of CPEB1 in liver cancer (*n* = 68, odd rows) and adjacent non-cancerous liver tissues (*n* = 60, even rows) in a tissue microarray (TMA), the staining scores of CPEB1 in liver cancer were lower than those observed in the adjacent normal liver tissues. Immunohistochemistry staining indicated that CPEB1 immunostaining primarily occurred in the cytoplasm of liver cancer tissues cells. **f** The expression levels of CPEB1 by immunostaining were moderately positive, weakly positive and negative in cancer samples, stronger staining was observed in peritumoral tissues. Left image, original magnification × 10; right image, magnification × 400; the white squares indicate the area shown at higher magnification. **g** Correlation analysis of CPEB1 expression with CD133 expression in 80 HCC specimens by qRT-PCR analysis in biopsies and immunohistochemistry staining in paraffin-embedded tissue. The data are presented as the means ± SEM. *P < 0.05

In mRNA expression data downloaded from TCGA, CPEB1 was found to be significantly downregulated in primary HCC tissues (normal, *n* = 50; primary tumor, *n* = 371) (Fig. 2a). CPEB1 was also downregulated in human HCC tumor tissue compared with adjacent non-tumor tissue (Fig. 2b–d). Immunohistochemical staining of a TMA showed that the expression of CPEB1 was weaker in liver cancer tissue than in non-cancerous tissue and primarily occurred in the cytoplasm of liver cancer tissues cells (tumor tissues, *n* = 68; peritumoral tissues, *n* = 60). Immunohistochemical staining scores of CPEB1 in liver cancer were lower than those observed in the adjacent normal liver tissues (Fig. 2e). The intensities of CPEB1 immunostaining were moderately positive, weakly positive and negative in cancer samples, stronger staining was observed in peritumoral tissues (Fig. 2f). Additionally, as shown in Table 2, the expression of CPEB1 in HCC tumor tissues was significantly correlated with age (*P* = 0.043) and clinical stage (*P* = 0.037). However, lower CPEB1 expression levels did not represent the poorer overall survival of HCC patients (*P* > 0.05), which may be related to the corresponding sample size and sample differences (Fig. S1). The relative proportion of CD133 expression was higher in HCC tissue samples expressing low levels of CPEB1 by quantitative reverse transcriptase-PCR (qRT-PCR) analysis in biopsies and immunohistochemistry staining in paraffin-embedded tissue (Fig. 2g). These results further substantiate those found in HCC cells, CPEB1 is downregulated in HCC tissue and this could be as a consequence of CSCs in the tumor tissue.

### Effects of CPEB1 overexpression and knockdown on cell self-renewal, migration and chemoresistance in vitro

**Fig. 3:**
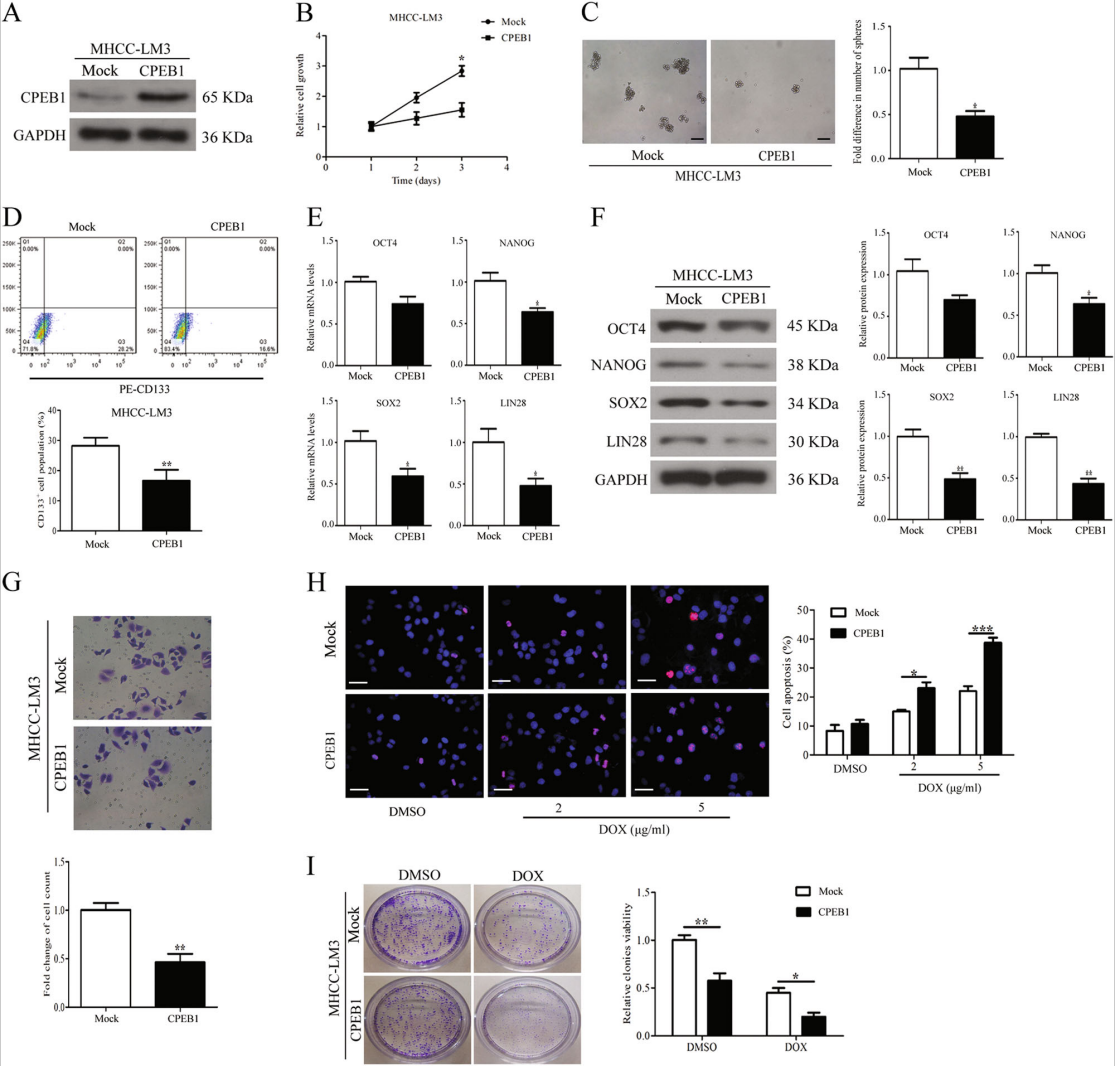
CPEB1 overexpression inhibits cell self-renewal, migration and chemoresistance. **a** CPEB1 was ectopically expressed in MHCC-LM3 cells and the CPEB1 expression levels were assessed by immunoblotting assays. **b** Cell viability was assessed using the CCK8 assay and significantly lower rates of cell proliferation were observed compared with the negative control. **c** By accessing the number and size of spheroids, spheroid formation ability in cells overexpressing CPEB1 was found to be weakened compared with the control. **d** CD133^+^ cell populations were decreased in MHCC-LM3 cells with CPEB1 overexpressed. **e**, **f** Expression of the major pluripotency factors OCT4, NANOG, SOX2 and LIN28 was decreased in CPEB1-expressing cells compared with control measured by quantitative RT-PCR and immunoblotting. **g** CPEB1 reduced cell migration property in a transwell assay detected by crystal violet staining. **h** Immunofluorescence staining for TUNEL (red) and nuclei were counterstained with DAPI (blue). Overexpression of CPEB1 promotes the dose-dependent apoptosis of doxorubicin compared with the control. **i** The indicated cells were treated with doxorubicin (5 μg/ml) or DMSO for 14 days and the cell viability of the cells was determined by a colony formation assay. Representative wells and results of at least three independent experiments are shown. The data are presented as the means ± SEM. *P < 0.05, **P < 0.01, ***P < 0.001

After establishing that CPEB1 expression may be reduced in CSCs, we assessed the outcome of overexpressing CPEB1 in MHCC-LM3 cells. CPEB1 was ectopically expressed in the MHCC-LM3 cell line, which showed moderate CPEB1 overexpression that was confirmed by immunoblotting (Fig. 3a). The results of the CCK8 assay showed that CPEB1 significantly inhibited HCC cell proliferation (Fig. 3b). The ability to form spheroids was also reduced in cells overexpressing CPEB1 compared with controls (Fig. 3c). CD133 is a functional liver CSC marker, we assessed the change of the CD133^+^ cell population in indicated cells. After overexpressing CPEB1, the CD133^+^ cell population decreased from 28.2% to 16.6% (Fig. 3d). The mRNA expression and protein levels of the major pluripotency factors OCT4, NANOG, SOX2 and LIN28 were all reduced in response to CPEB1 overexpression, significantly for NANOG, SOX2 and LIN28 but not for OCT4 expression. (Fig. 3e, f). In addition, cell migration was also obviously inhibited (Fig. 3g). Moreover, overexpression of CPEB1 in cells accelerated a dose-dependent doxorubicin-induced apoptosis, and further reduced cell viability after doxorubicin treatment compared with the control (Fig. 3h, i).

**Fig. 4:**
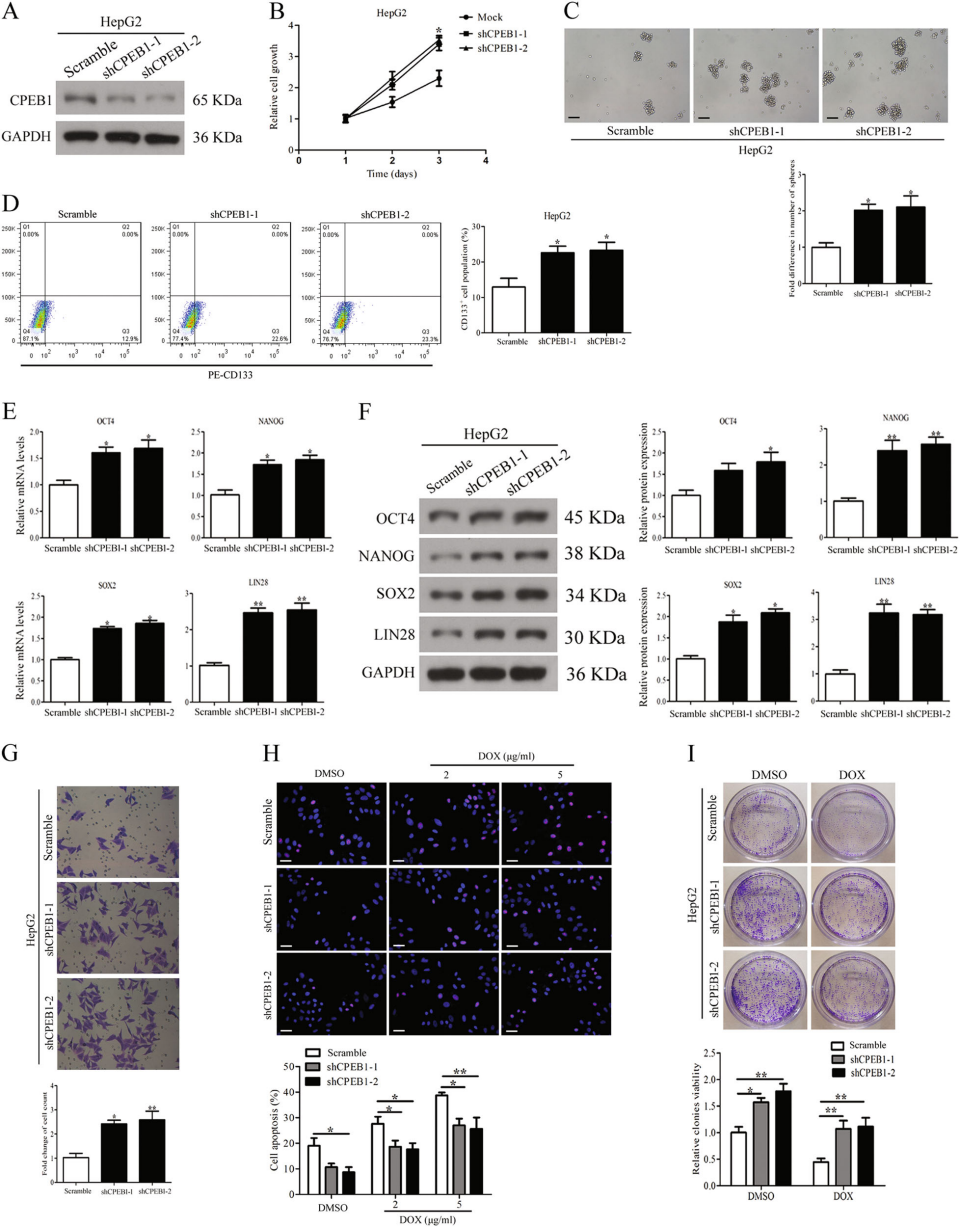
CPEB1 knockdown promotes cell self-renewal, migration and chemoresistance in vitro. **a** The knockdown efficiency of CPEB1 in HepG2 cell lines was verified by immunoblotting. **b** CPEB1 shRNA was stably expressed in HepG2 cell lines, and cell viability was assessed using the CCK8 assay and showed significantly higher rates of cell proliferation compared with negative control (Mock). **c** Comparison of spheroid formation ability among indicated cells by accessing the number and size of spheroids. **d** CD133^+^ cell populations were increased in CPEB1-shRNA expressing cells vs control-shRNA cells. **e**, **f** silencing of CPEB1 enhanced the expression of OCT4, NANOG, SOX2 and LIN28 in indicated cells detected by quantitative RT-PCR and immunoblotting assays. **g** CPEB1 knockdown promotes cell migration detected by a transwell assay detected by crystal violet staining. **h** Immunofluorescence staining for TUNEL (red) and nuclei were counterstained with DAPI (blue). CPEB1 knockdown impaired dose-dependent apoptosis of doxorubicin (2 and 5 μg/ml) compared with control-shRNA in HepG2 cells. **i** CPEB1-shRNA expressing cells and control cells were treated with doxorubicin (5 μg/ml) or DMSO for 14 days and the cell viability of the cells was determined by colony formation assay. Each experiment was performed in triplicate. The data are presented as the means ± SEM. *P < 0.05, **P < 0.01

We then assessed the effects of silencing CPEB1 on cell stemness, migration and chemoresistance. CPEB1 expression levels were assessed by immunoblotting (Fig. 4a). Silencing of CPEB1 significantly increased HCC cell viability, as determined by the CCK8 assay (Fig. 4b). Spheroid formation (Fig. 4c) and the CD133^+^ cell population (Fig. 4d) were promoted by CPEB1 knockdown, and the expression of OCT4, NANOG, SOX2 and LIN28 was also increased in indicated HepG2 cells (Fig. 4e, f). HepG2 cells were transfected with an empty vector as a control. Silencing of CPEB1 also promoted cell migration by transwell assay (Fig. 4g). Moreover, CPEB1 knockdown increased HCC cells resistance to doxorubicin, whereas apoptosis was decreased (Fig. 4h, i).

Overall, the results indicate that when CPEB1 is overexpressed in HCC cells, self-renewal, migration and chemoresistance is inhibited, whereas when CPEB1 is knocked down in HCC cells, self-renewal, migration and chemoresistance are promoted.

### CPEB1 regulates the poly(A) tail length and translation of SIRT1 mRNA

**Fig. 5:**
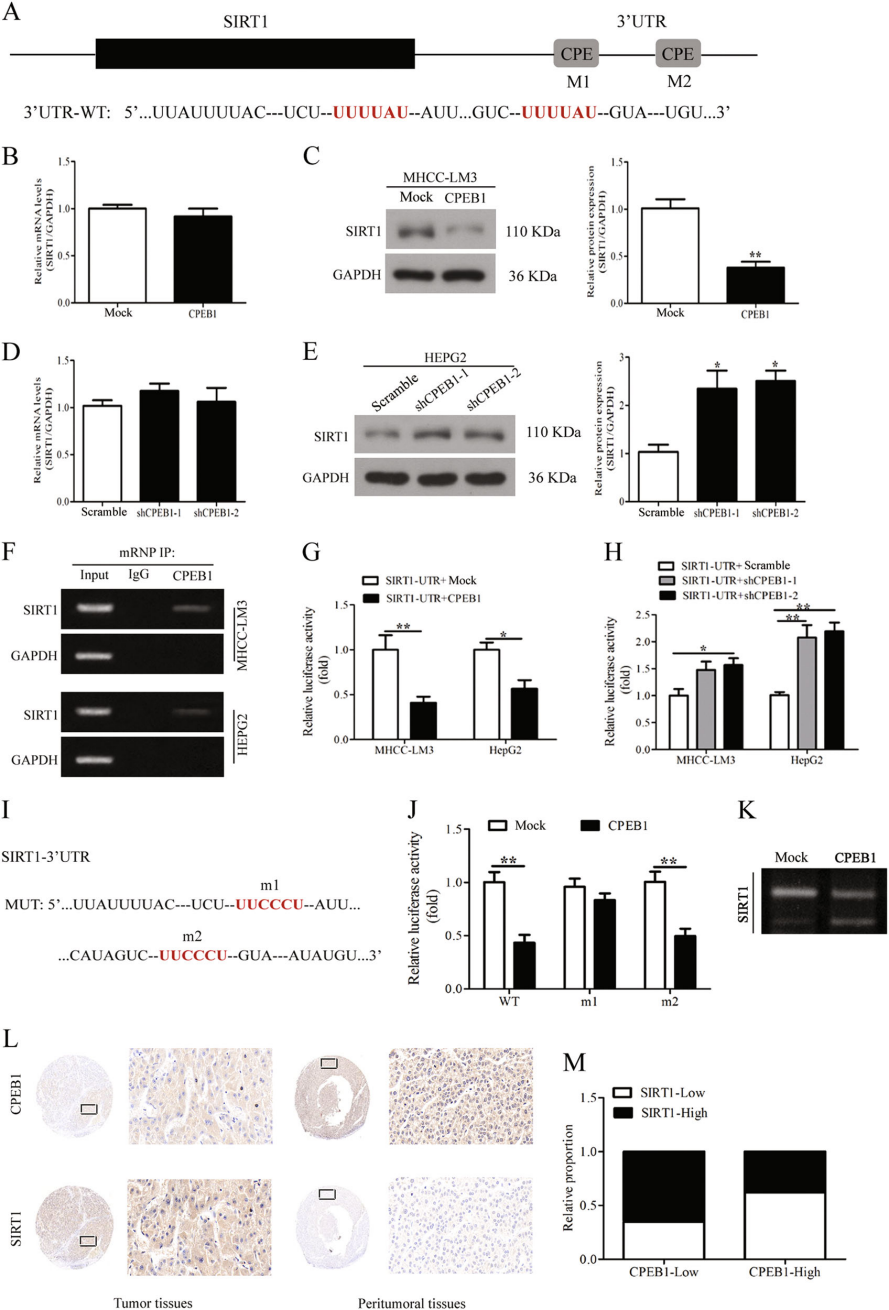
CPEB1 directly regulates SIRT1 by controlling translation. **a** SIRT1 mRNA 3′-UTR putative sites targeted by CPEB1 (red). **b**, **c** SIRT1 protein levels were suppressed in HCC cells overexpressed CPEB1 and elevated after knockdown of CPEB1 in the HepG2 cells (**d**, **e**) but not mRNA levels, analyzed by immunoblotting and quantitative RT-PCR. **f** A RIP assay was carried out to detected the binding of CPEB1 and SIRT1. SIRT1 and GAPDH mRNA levels were detected using RT-PCR as shown in the representative cropped gel. **g** SIRT1-UTR luciferase and Renilla luciferase constructs were co-transfected with an empty vector (Control) or CPEB1. Relative luciferase activity is the ratio between firefly luciferase and Renilla control luciferase. **h** SIRT1-UTR luciferase constructs were co-transfected with shRNA negative control (Control) or shCPEB1-1/2 against CPEB1, and cells were treated as in (**g**). **i**, **j** To verify if the two putative CPE were functional, we mutated the SIRT1-UTR reporter construct at either the first or second CPE site (m1 and m2, respectively). Relative luciferase activity showed that m1 is not sensitive to the action of CPEB1, whereas m2 behaved as the WT construct. **k** MHCC-LM3 cells were transfected as indicated in the panels and after 48 h, total RNA was isolated and subjected to PAT assays. **l** Representative immunohistochemistry images of CPEB1 and SIRT1 in the same views of HCC samples in a TMA and **m** a correlation analysis of CPEB1 expression with SIRT1 expression in 80 HCC specimens. The data are presented as the means ± SEM. *P < 0.05, **P < 0.01

SIRT1 is a NAD^+^-dependent histone deacetylase that modifies proteins through deacetylation and is associated with several carcinomas^26^, we discovered that the 3′-UTR of SIRT1 mRNA harbors two putative CPE sequences as shown in Fig. 5a. To check if CPEB1 could affect SIRT1 expression, we assessed the possibility that CPEB1 regulates SIRT1 in HCC cells. Overexpression of CPEB1 was found to suppress SIRT1 protein levels but mRNA levels were unchanged (Fig. 5b, c). CPEB1 knockdown elevated SIRT1 protein levels but had no obvious effect on mRNA levels (Fig. 5d, e), indicating that CPEB1 action is exerted through the modulation of SIRT1 mRNA. To further investigate the interaction between CPEB1 and SIRT1, a RIP assay was carried out. The results confirmed that CPEB1 was able to bind to SIRT1 mRNA (Fig. 5f). Luciferase assays were used to measure SIRT1 activity in MHCC-LM3 and HepG2 cells with CPEB1 overexpressed or knocked down. SIRT1-UTR luciferase activity was reduced in cells with CPEB1 overexpressed compared with control cells. However, SIRT1-UTR luciferase activity was significantly higher in cells with CPEB1 knocked down compared with control cells (Fig. 5g, h). To further verify if the identified CPE sites were functional with SIRT1, we mutated both sites (m1 and m2) in SIRT1-UTR reporter constructs (Fig. 5i). Relative luciferase activity showed that the mutated m1 CPE site was insensitive to the action of CPEB1, whereas m2 gave the same results as the WT construct, which indicates that the m1 CPE site interacts with CPEB1 (Fig. 5j). As CPEB1 was originally identified as a polyadenylation factor and as polyadenylation strongly stimulates translation, we asked whether the polyadenylation status of SIRT1 mRNA was altered by CPEB1 modulation. A PAT assay was performed on both endogenous SIRT1 mRNA derived from CPEB1-transfected cells or controls (Fig. 5k). The results showed that indeed SIRT1 mRNA underwent poly(A) tail shortening upon CPEB1 overexpression. Moreover, the regulation is further validated in HCC tumor and peritumoral tissues, a lower level of SIRT1 protein corresponded with a higher level of CPEB1 in the same visual field of peritumoral tissues, and vice versa in the HCC tumor tissues (Fig. 5l). Moreover, CPEB1 expression was correlated with SIRT1 expression in 80 HCC specimens (Fig. 5m). These results indicate that CPEB1 regulates the polyadenylation and translation of SIRT1 mRNA in HCC cells possibly by interacting with a CPE site.

### SIRT1 impaired the suppression of cell self-renewal, migration and chemoresistance induced by CPEB1 overexpression

**Fig. 6:**
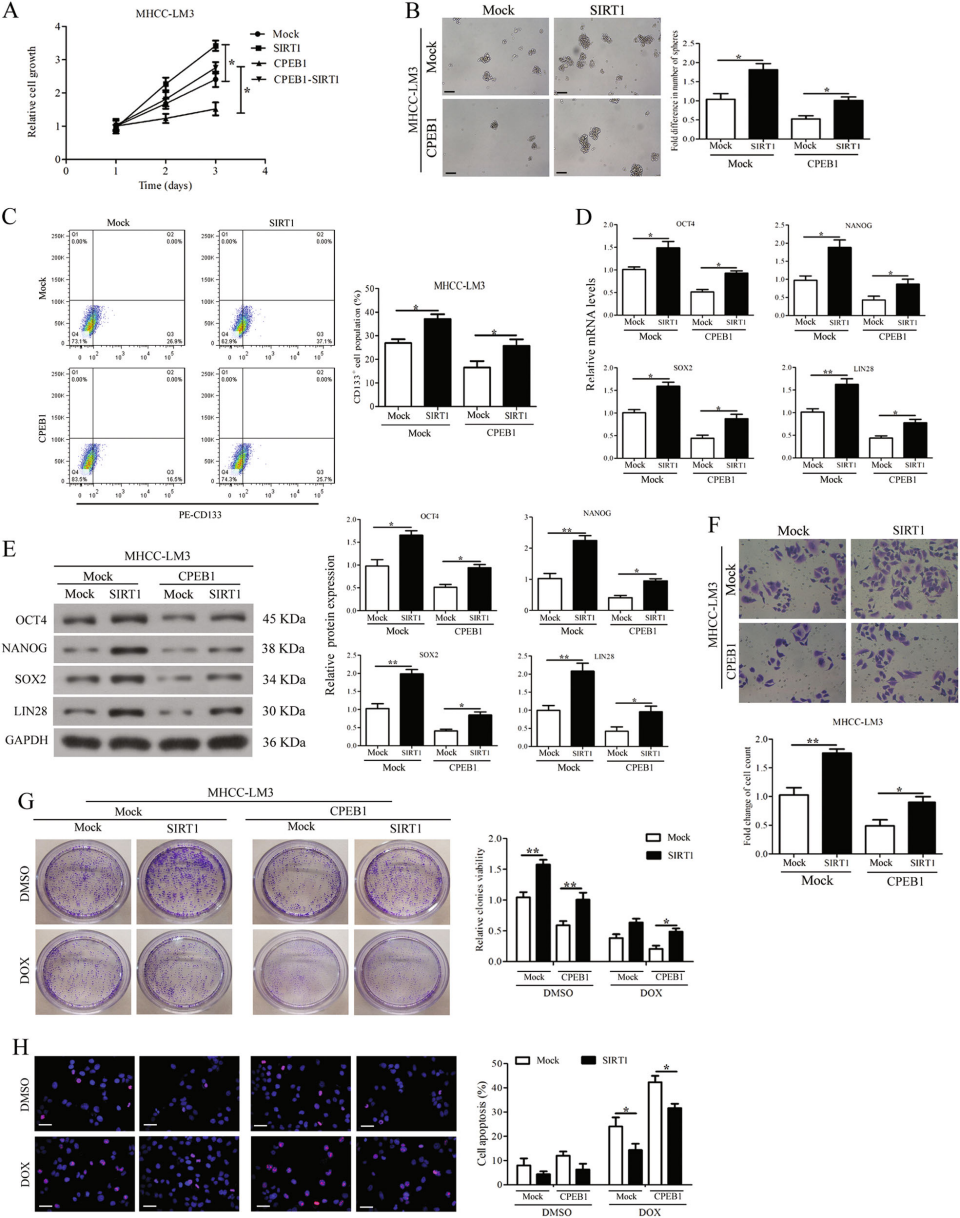
SIRT1 impaired the suppression of cell self-renewal, migration and chemoresistance induced by CPEB1 overexpression. **a** CCK8 assay results showed overexpressing SIRT1 significantly promotes HCC cell viability compared with negative control (Mock) and weakened the suppression in MHCC-LM3 cells induced by co-transfecting with CPEB1vector. **b** Comparison of spheroid formation ability among indicated cells by accessing the number and size of spheroids. **c** SIRT1 overexpression significantly impaired the decrease in CD133^+^ cell populations induced by ectopic expression of CPEB1 in MHCC-LM3 cells vs control. **d**, **e** SIRT1 impaired the suppression on the expression of OCT4, NANOG, SOX2 and LIN28 in indicated cells assessed by quantitative RT-PCR and immunoblotting assays. **f** SIRT1 overexpression significantly attenuated the inhibition of cell migration in CPEB1-expressing cells detected by transwell assay using crystal violet staining. **g** SIRT1/control vector was co-transfected in CPEB1-expressing cells or control cells. Cells were treated with doxorubicin (5 μg/ml) or DMSO for 14 days as indicated and the cell viability of the cells was determined by colony formation assay. Each experiment was performed in triplicate. **h** Immunofluorescence staining for TUNEL (red) and nuclei were counterstained with DAPI (blue). CPEB1 promoted dose-dependent apoptosis of doxorubicin (2, 5 μg/ml) compared to Mock in MHCC-LM3 cells. The data are presented as the means ± SEM. *P < 0.05, **P < 0.01

To further characterize SIRT1, we assessed whether its overexpression influenced the cell functions in cells overexpressing CPEB1. Ectopically expressing SIRT1 significantly enhanced cell viability and weakened the suppression induced by CPEB1 overexpression in the MHCC-LM3 cell lines (Fig. 6a). We found that SIRT1 overexpression also significantly attenuated the inhibition of spheroid formation in cells overexpressing CPEB1 (Fig. 6b). Whereas SIRT1 overexpression increased the number of cells that were positive for the hematopoietic stem cell marker CD133 in a population of MHCC-LM3 cells overexpressing CPEB1 in which levels of CD133-positive cells had been reduced (Fig. 6c). The mRNA expression and protein levels of the major pluripotency factors OCT4, NANOG, SOX2 and LIN28 were significantly increased when SIRT1 was overexpressed but they were reduced by CPEB1 overexpression (Fig. 6d, e). The suppression of cell migration induced by CPEB1 overexpression was also impaired after co-transfecting with the SIRT1 vector (Fig. 6f). In addition, the resistance to doxorubicin (5 μg/ml) measured by cell apoptosis and colony formation was increased by SIRT1 overexpression in cells co-transfected with CPEB1 (Fig. 6g, h). These results indicate that overexpressing SIRT1 can attenuate the effects of overexpressing CPEB1 and increase the level of stem cell characteristics in HCC cells.

### Upregulation of CPEB1 reduces tumor growth, self-renewal and the chemoresistance of HCC cells in vivo

**Fig. 7:**
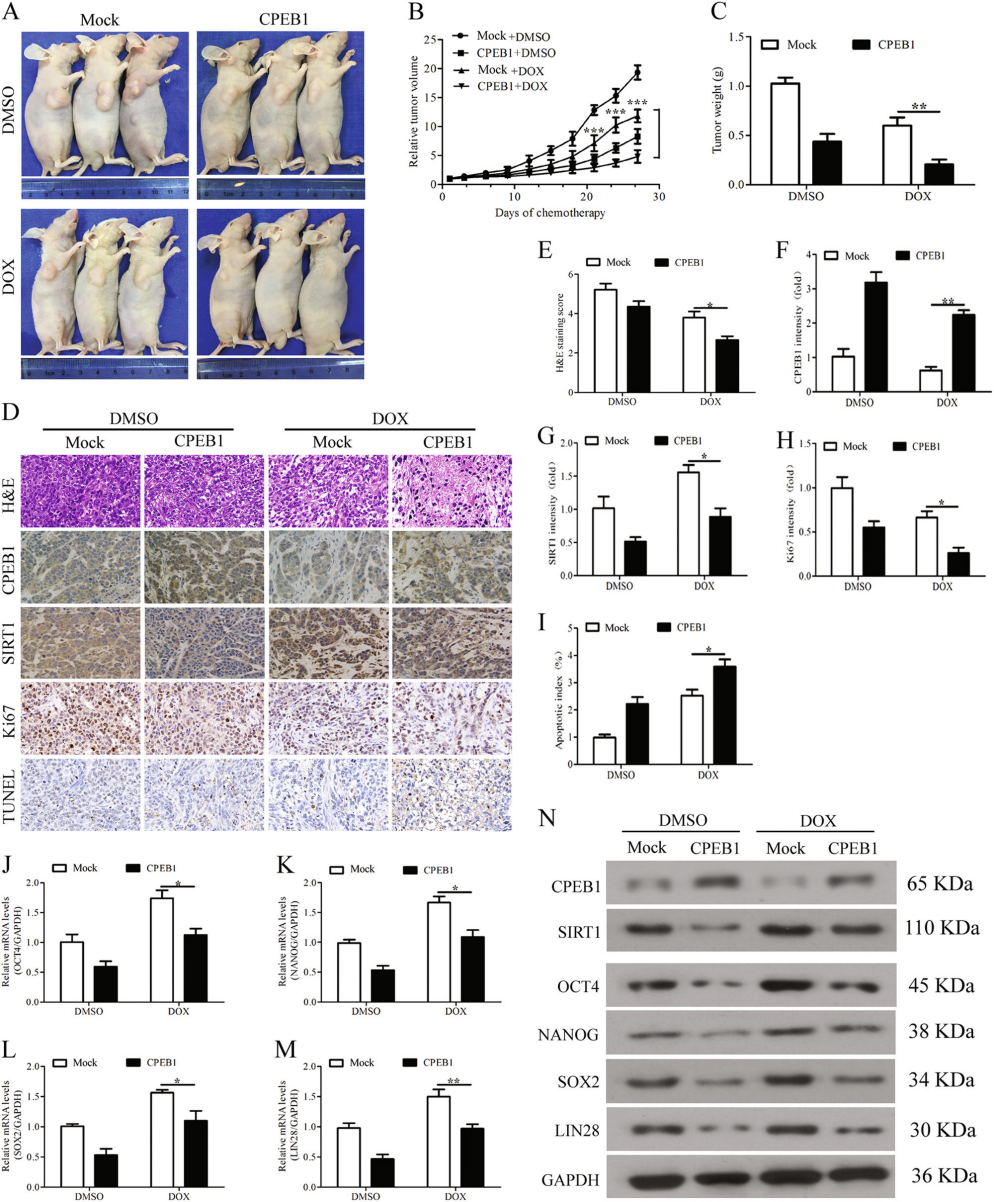
Upregulation of CPEB1 attenuates tumor growth, self-renewal and chemoresistance of HCC cells in vivo. **a–c** Tumor growth was assessed in nude mice that were subcutaneously injected into the right axilla with 1 × 10^6^ MHCC-LM3 cells with or without stable CPEB1 expression. After 2 weeks, doxorubicin (1 mg/kg) or DMSO was orally administered to the mice of each subgroup every 2 days for six doses (*n* = 6 per group). Subsequently, the xenograft tumor size was monitored every 3 days (volume = width^2^ × length × 1/2). Points represent the mean tumor volumes of three independent experiments. After 27 days, the xenograft tumors were excised from the nude mice, and CPEB1 inhibited the xenograft tumors growth (**b**, **c**). **d–i** Cancer cell morphology and apoptosis, and levels of CPEB1, Sirt1 and Ki-67 in the xenograft tumors were examined by H&E, TUNEL and immunohistochemical staining, and quantified with Image ProPlus (IPP) software (**j–n**). The levels of the major pluripotency factors OCT4, NANOG, SOX2 and LIN28 were detected in the xenograft tumors by quantitative RT-PCR and immunoblotting assays. The data are presented as the means ± SEM. *P < 0.05, **P < 0.01

After acquiring evidence that CPEB1 could inhibit cell self-renewal and chemoresistance in vitro, we next assessed whether it could replicate these results in vivo using nude mice. Twenty-four mice were randomly divided into two groups, and then subcutaneously injected in the right axilla with MHCC-LM3 cells with or without the expression of CPEB1. After 2 weeks, doxorubicin (1 mg/kg) or DMSO was orally administered once every 2 days for six doses (*n* = 6 per group). Tumors from mice that received MHCC-LM3 cells expressing CPEB1 were significantly smaller than those receiving control MHCC-LM3 cells (Fig. 7a–c). Moreover, MHCC-LM3 cells expressing CPEB1 were more susceptible to doxorubicin. Cancer cell morphology was less pronounced in tumor cells expressing CPEB1 and apoptosis was increased (Fig. 7d). Levels of Sirt1 and Ki-67 activity were reduced in cells overexpressing CPEB1 (Fig. 7e–i). The levels of OCT4, NANOG, SOX2 and LIN28 in xenograft tumors expressing CPEB1 were significantly reduced in response to doxorubicin compared with control cells (Fig. 7j–n). Overall, these results indicate that the upregulation of CPEB1 in HCC cells reduces tumor growth, self-renewal and chemoresistance in a mouse model.

## Discussion

Increasing evidence suggests that a subpopulation of cells that exhibit stem cell properties may give rise to HCC and lead to an increase in proliferation, metastasis and chemoresistance^27–29^. Furthermore, a subpopulation of cells in the human HCC cell lines MHCC97-H, MHCC97-L, Huh7 and HCCLM3 were found to initiate tumorigenesis when grafted into mice^9^. Therefore, finding a way to regulate subpopulations of CSCs in HCC could lead to an improved response to therapy. In this study, we found that CPEB1, a CPE-binding protein involved in the regulation of mRNA translation, negatively mediates HCC cancer stemness and chemoresistance in vitro and in vivo. Analysis of CPEB1 expression in HCC showed that it was expressed at lower levels in HCC tissues and cell lines than in adjacent non-tumor tissues and normal hepatic cells. Overexpression or silencing of CPEB1 in cells regulated mRNA translation to inhibit or promote cell self-renewal, metastasis and chemoresistance.

In the present study, we discovered CPEB1, as a polyadenylation factor, may suppress the polyadenylation and translation of SIRT1 in HCC cells, which has not yet been identified. In addition, we confirmed whether CPEB1 could bind to SIRT1 by performing a RIP assay and also assessed changes in the behavior of SIRT1 after mutating the CPE sites in CPEB1. Moreover, we found that CPEB1 overexpression could shorten the poly(A) tail of SIRT1 mRNA via a PAT assay. Our results indicate that the expression of CPEB1 in CSCs was negatively correlated with cancer progression, which is similar to results in other cancers^23,30^. Furthermore, overexpressing SIRT1 increased spheroid formation, self-renewal, chemoresistance and significantly weakened the inhibition of cell migration in cells expressing CPEB1. However, the role of SIRT1 in cancer progression has been controversial, with some studies reporting a tumorigenesis function and others reporting a tumor-suppressor function^31^. A Sirt1^−/−^ mouse model indicated that SIRT1 prevented the induction of prostate intraepithelial neoplasia and inhibits reactive oxygen species production by promoting mitophagy and a low level of SIRT1 has been associated with decreased recurrence-free survival in prostate cancer, which implies that SIRT1 could have a defensive role against cancer^32^. However, a number of studies have found that SIRT1 is overexpressed in cancer cells^33^, which would imply that it may have an oncogenic role. In addition, SIRT1 has been found to be associated with a poor prognosis in HCC and its interaction with lncRNA HULC is thought to increase chemoresistance in HCC by promoting autophagy^34,35^. SIRT1 is known to interact with several pathways and, therefore, it has been suggested that the status of the proteins in these pathways could determine whether it behaves as an oncogene or tumor suppressor^31^. In our study, we concentrated on the role of SIRT1 in CSCs, which may be another reason for the conflicting reports about its role in tumorigenesis, other studies may report its status in somatic cells. A similar study to ours demonstrated that SIRT1 mediates the self-renewal and tumorigenicity potential of liver CSCs through an interaction with SOX2^34^, which may also be involved in the regulation of CPEB1 as some results have shown in this study. SIRT1 was also thought to regulate the transcription of SOX2 by chromatin-based epigenetic changes^34^. In addition, SIRT1 deacetylates YAP2 protein in HCC cells and SIRT1-mediated deacetylation increases the YAP2/TEAD4 association, leading to YAP2/TEAD4 transcriptional activation and upregulated cell growth and enhances the chemosensitivity of HCC cells^36,37^. It also mediated FoxO1 deacetylation and regulated multidrug resistance-associated protein 2 expression to enhance the chemosensitivity of breast cancer cells^26^. The speculation that the above pathways or genes may also be associated with the regulation of CPEB1 in HCC cells should be validated, and more relevant pathways may be further studied for this research in the future.

To conclude, we analyzed the influence of CPEB1 on cancer stemness in HCC cells by analyzing self-renewal ability, chemoresistance, metastasis and expression of the stem cell-related genes OCT4, NANOG, SOX2, LIN28, CD24, EpCAM and CD133. We found that CPEB1 is downregulated in HCC, which supports the results of other studies. Altering the expression of CPEB1 influences the stemness of HCC cells. Overexpression reduces stemness, whereas inhibiting CPEB1 expression increases it. Mechanistically, we investigated whether CPEB1 could directly target the 3′-UTR of SIRT1 and established that it does by performing a coimmunoprecipitation assay. Moreover, CPEB1 appears to regulate the polyadenylation and translation of SIRT1 to mediate cancer stemness in vitro and in vivo. Taken together, our findings suggest that the RNA-binding protein CPEB1 plays a potentially key role in CSC regulation and tumor growth. Moreover, because RNA processing activities are important for normal tissue development and stem cell self-renewal, RNA-binding proteins or their regulatory circuits may become prime therapeutic targets whose neutralization may be effective in blunting the driving force of a broad range of malignancies, particularly solid tumors whose treatment remains a major challenge.

## Electronic supplementary material

The relationship between CPEB1 expression levels and overall survival of HCC patients

Figure S1
