# Supplementary figures and images for: CPEB1 mediates hepatocellular carcinoma cancer stemness and chemoresistance
Source: Cell Death Dis. 2018 Sep 20;9(10):957. doi: 10.1038/s41419-018-0974-2 (PMC6148052; doi:10.1038/s41419-018-0974-2)

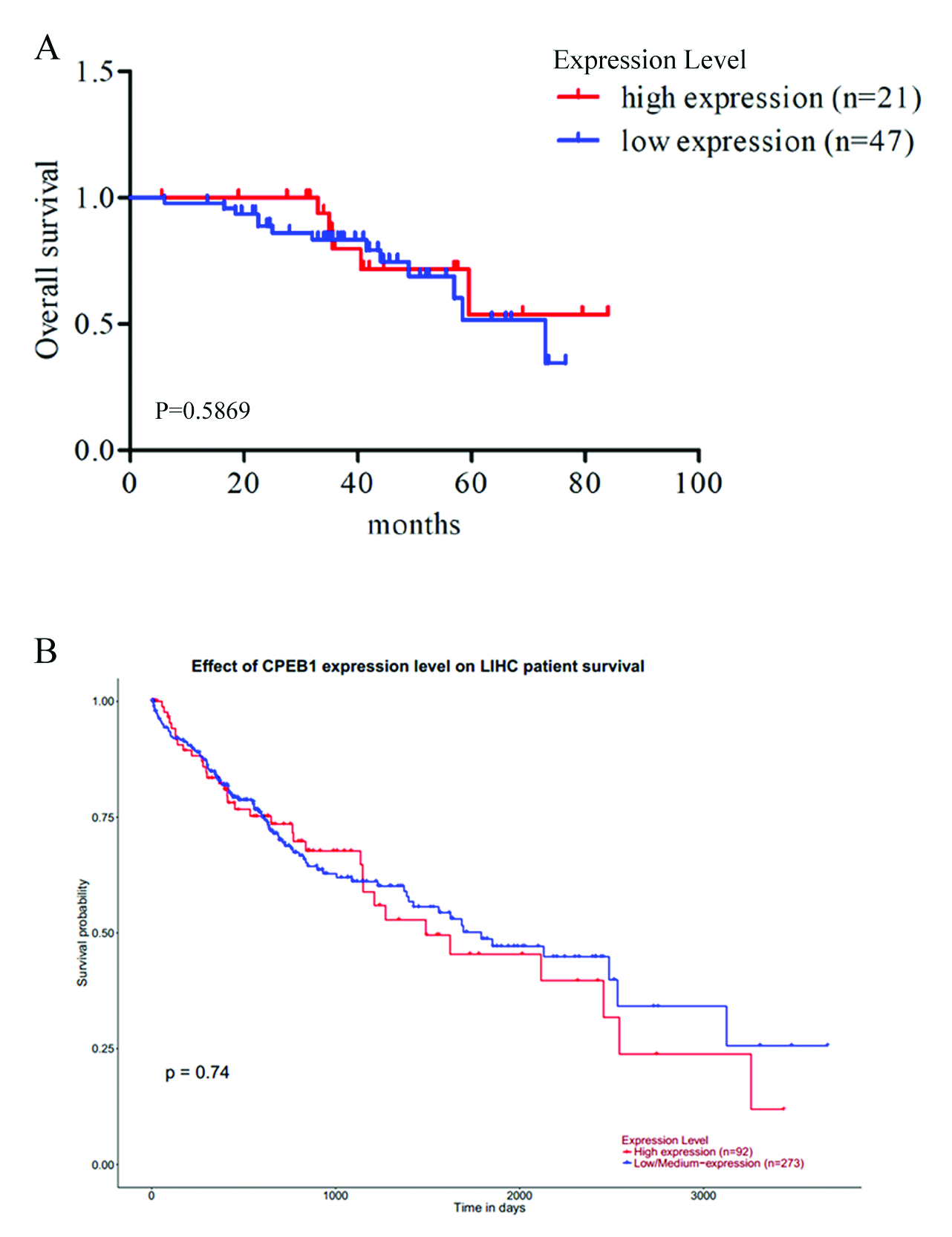

Supplement: Supplementary file 1 — The relationship between CPEB1 expression levels and overall survival of HCC patients [file 41419_2018_974_MOESM1_ESM.tif]
