# Supplementary material for: CPEB1 mediates hepatocellular carcinoma cancer stemness and chemoresistance
Source: Cell Death Dis. 2018 Sep 20;9(10):957. doi: 10.1038/s41419-018-0974-2 (PMC6148052; doi:10.1038/s41419-018-0974-2)
Supplement: Supplementary file 2 — Figure S1 [file 41419_2018_974_MOESM2_ESM.docx]

Figure S1. (A) Kaplan–Meier analysis of overall survival in 68 HCC patients according to CPEB1 expression. (B) Effect of CPEB1 expression level on LIHC patient survival in data from TCGA.
